# Prevalence, Diagnostic Accuracy, and Antimicrobial Resistance Patterns of Uropathogens in Urinary Tract Infections at a Health Facility in Kumasi, Ghana

**DOI:** 10.1002/mbo3.70028

**Published:** 2025-08-27

**Authors:** Michael Nana Antwi‐Boasiako, Frederick Ayensu, Aaron Awuah, Abdul‐Hakim Mutala, Kwadwo Boampong, Kingsley Badu

**Affiliations:** ^1^ Department of Theoretical and Applied Biology Kwame Nkrumah University of Science and Technology Kumasi Ghana; ^2^ HopeXchange Medical Centre Kumasi Ghana

**Keywords:** dipstick, microscopy, susceptibility, urinary tract infection, urine culture

## Abstract

Urinary tract infections (UTIs) are one of the most prevalent infections in both men and women. The most common causative organisms are *Escherichia coli, Klebsiella* spp., and *Staphylococcus* spp. UTIs are diagnosed using various diagnostic methods with urine culture as the gold standard. This study aimed to estimate the prevalence of UTIs, compare the tools employed in the diagnosis of UTIs, and assess the antibiotic susceptibility pattern of bacterial isolates among patients in a local health facility in Kumasi, Ghana. A cohort of 208 participants were enlisted for a cross‐sectional study at the HopeXchange Medical Centre, Kumasi. Mid‐stream urine samples were collected from participants and analyzed by culture, microscopy, and dipstick methods. Culture isolates were tested on Mueller‐Hinton Agar applying the Kirby Bauer method against a range of antibiotics. The prevalence of *Staphylococcus* spp. (25%) and *Klebsiella* spp. (16.7%) were higher than that of *Escherichia coli* (8.3%). UTI was more common in individuals between 50 and 59 years of age (36.5%) and mostly associated among women (76.9%). Overall, culture confirmed UTI prevalence was 23.08% while dispstick and microscopy diagnosed 44.23% and 47.60% of UTIs, respectively. Microscopy exhibited a sensitivity of 68.8% and specificity of 58.8% while the dipstick had a sensitivity of 60.4% and specificity of 60.6% compared to urine culture. Meropenem showed 100% susceptibility, while high resistance was observed for chloramphenicol and ceftriaxone against *Escherichia coli, Staphylococcus* spp*., Providencia* spp., and *Klebsiella* spp. UTI was significantly associated with age, gender, and the standard diagnostic method (*p* < 0.05).

## Graphical abstract

The prevalence of urinary tract infections, and antibiotic susceptibility pattern of the bacterial isolates were investigated among patients. The findings suggest an increasing rate of antibiotics resistance among the bacterial isolates, and a high prevalence rate of UTIs among women. The bacterial isolates were all found to be susceptible to meropenem.

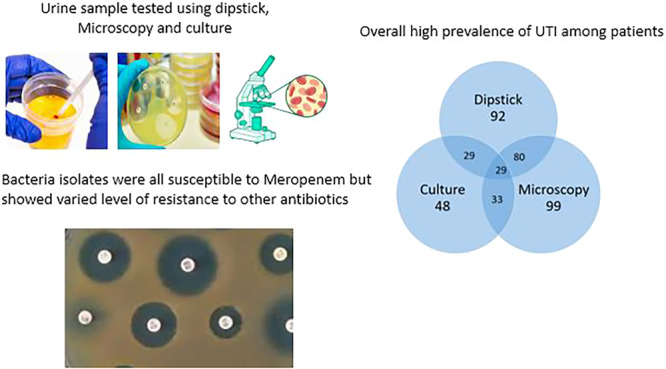

## 1 Introduction

Annually, urinary tract infections (UTIs) and their associated complications impose a substantial global burden, accounting for an estimated 404.61 million cases, 236,790 deaths, and 520,200 disability‐adjusted life years (Yang et al. 2022). Particularly, clinicians in developing nations bear the brunt of this health challenge, encountering UTIs at an alarming rate, with approximately 8.3 million healthcare visits attributed to UTIs each year (Linhares et al. 2013).

Infection of the urinary tract arises from the invasion of microorganisms within the urinary tract (Prakash and Saxena 2013). UTIs pose a risk to individuals of all demographics; however, the likelihood of infection varies with factors such as age, gender, and various predisposing conditions. Notably, UTIs are more common in women than men (Dielubanza and Schaeffer 2011). This observed gender disparity in UTI prevalence is due to the structural differences in the urinary tract anatomy of males and females as females typically possess shorter urethras, measuring between 3 and 4 cm in length, in contrast to males, whose urethras are approximately 20 cm long (Hooton 2012). Additionally, factors such as poor hygienic practices, fecal contamination, sexual activity, and other related variables contribute to the heightened vulnerability of women to UTIs (Hooton 2012).

The natural flow of urine from the bladder also serves as a mechanism to expel bacteria from the body; however, despite this inherent defense, UTIs and the development of antimicrobial resistance (AMR) of causative agents persist (Iregbu and Nwajiobi‐Princewill 2013). *Escherichia coli* stands out as a prominent bacterium responsible for the majority of UTIs. This microorganism exerts its pathogenicity by adhering to, colonizing, and proliferating within the umbrella cells of the bladder epithelium (Iregbu and Nwajiobi‐Princewill 2013; Battikhi and Battikhi 2015). The ability of *E. coli* to navigate the urinary system is facilitated by bacterial motility, enabling it to ascend and potentially obstruct the urinary tract's proper flow (Lane and Takhar 2011).

Studies conducted in Ghana have reported varying prevalence rates of UTIs: 86% among hospitalized patients (Gambrah et al. 2021), 42.75% among pregnant women (Forson et al. 2018), and 15.9% in the general population (Afriyie et al. 2015). Additionally, the prevalence of multi‐drug resistant (MDR) UTIs has been reported at 93.6% (Donkor et al. 2019). UTIs have a significant impact on public health and economic development in Ghana, contributing to increased healthcare expenditures, reduced productivity, and potential mortality from complications such as sepsis (Opare‐Asamoah et al. 2025; Gambrah et al. 2021; Donkor et al. 2019).

Ghana has developed a national policy and action plan aligned with the World Health Organization's Global Action Plan on AMR, recognizing AMR as a critical public health threat (Yevutsey et al. 2017). A nationwide study investigating AMR trends among different uropathogens over time and across various regions of Ghana would strengthen the evidence base for informed policy‐making. Such research would support the five strategic objectives of the WHO Global Action Plan on AMR: awareness, surveillance, infection prevention and control, responsible antimicrobial use, and research and innovation (World Health Organisation 2023).

The emergency department frequently serves as the primary locus for UTI presentations, where prompt diagnostic assessments through urinalysis are commonplace (Gordon et al. 2013). It is paramount to underscore that diagnosing a UTI should not rely solely upon urinalysis findings; instead, a comprehensive evaluation encompassing the patient's medical history and subsequent urine culture results assumes critical significance. In a recent investigation, it was revealed that within a cohort comprising 153 women aged 70 years and older, who received a UTI diagnosis in the emergency department, microbiological confirmation of a UTI was absent in 43% of cases. Remarkably, a substantial 95% of these culture‐negative cases had been administered antibiotic therapy (Gordon et al. 2013). Accurate diagnosis will reduce the morbidity and mortality attributable to UTIs, in addition to reducing the over‐prescription of antibiotics (Chu and Lowder 2018).

Many diagnostic methods, including wet mount microscopy, Gram staining, dipstick analysis, and automated tests, are available for the assessment of UTIs. However, it is essential to recognize that the gold standard for the diagnosis of UTIs remains the quantitative urine culture, as emphasized by Perkins et al. (2012). Given the potentially severe consequences of delayed UTI diagnoses, especially in pregnant women, early detection within a laboratory setting through means such as dipstick test, microscopic examination, or urine culture is of paramount importance (Chu and Lowder 2018). The threshold for diagnosing a UTI is predicated on the quantity of bacteriuria present, that is, the presence of approximately 100,000 (1 × 10^5^) CFU/mL. However, it is noteworthy that in specific populations, particularly those displaying pronounced symptomatic presentations, 100 (1 × 10^2^) CFU/mL may be considered as a positive diagnostic indicator (Giesen et al. 2010). The development of uropathogens that are resistant to the commonly used antibiotics has made UTIs more complicated and challenging to treat (Gessese et al. 2017).

This study sought to estimate the prevalence of UTIs, determine the risk factors associated with UTIs, evaluate the accuracy of diagnostic tools employed in the confirmation of UTIs, and assess the antibiotic susceptibility pattern of uropathogens isolated from patient samples.

## 2 Materials and Methods

### 2.1 Study Site and Design

The research was conducted at the HopeXchange Medical Centre, which is a mission hospital situated in Kumasi in the Ashanti Region of Ghana. UTI has been among the top 10 infectious diseases from 2018 to 2021 at HopeXchange Medical Centre (2022). The study was carried out over the course of 3 months, from July to September 2022. The hospital is located within the coordinates 6°39′N latitude and 1°39′W longitude. A cross‐sectional study design was employed for this study to assess the prevalence of UTI among patients.

### 2.2 Sample Collection

Utilizing Cochran's method and a prevalence of 10.1% (Donkor et al. 2019), the sample size was determined as

$$n=\frac{z^{2}p(1-p)}{d^{2}}$$

where *n* is the sample size, *p* the prevalence rate, *z* represents the standard normal distribution's critical value at the 5% level (1.96), and *d* the allowable sampling error (0.05).

A total of 208 patients were recruited as participants for this study. The study employed a consecutive sampling technique, where all eligible participants who met the inclusion criteria were enrolled sequentially until the target sample size was achieved. This method minimized selection bias by ensuring that every eligible participant has an equal chance of being included, without subjective selection by the researchers. Patients, who were within the ages of 18 and 59 years inclusive and of either sex, were included in the study because hormonal changes and other lifestyle choices make this age group especially vulnerable to UTIs (Flores‐Mireles et al. 2015; Storme et al. 2019). Individuals who had been administered antibiotics within the preceding 2 weeks or those who declined to provide informed consent for the study were excluded. Subsequently, patients who consented to the study were provided with an appropriately labelled sterile container and guided on how to obtain a clean‐catch, mid‐stream urine sample. Samples were transported to the microbiology laboratory within the hospital for analysis. Demographic information of study participants was collected through questionnaire administration.

### 2.3 Culturing and Identification of Isolates

Mid‐stream urine samples were aseptically plated onto cysteine lactose electrolyte‐deficient (CLED) agar plates, specifically chosen for their proficiency in supporting the growth of a diverse spectrum of potential urinary pathogens. Plates were incubated for 18 to 24 h at 37°C. Bacterial colonies were enumerated, and substantial bacteriuria was defined as counts equal to or exceeding 1 × 10^5^ CFU/mL. To confirm the presence and identification of bacterial species, different methods were employed. These included the examination of morphological characteristics of the bacterial colonies, Gram‐stain reactions, and biochemical assays such as catalase, coagulase, urease, triple sugar iron (TSI), indole, oxidase, glucose fermentation, and citrate tests.

For *E. coli*, the isolates were Gram‐negative, lactose fermenting, indole‐positive, citrate‐negative, methyl red positive, and urease‐negative. They also produced acid and gas on glucose fermentation in TSI with no H₂S production.

For *Klebsiella* spp., the isolates were Gram‐negative rods, lactose fermenters, indole‐negative, citrate‐positive, urease‐positive, nonmotile, and showed gas production on TSI with no H₂S.

For *Providencia* spp., the isolates were Gram‐negative rods, non‐lactose fermenting, indole‐positive, citrate‐positive, urease‐positive, and produced an alkaline/acid reaction on TSI without gas or H₂S. They were also motile and oxidase‐negative.

Oxidative test was used to differentiate *Staphylococcus* spp. from *Micrococcus* spp. An oxidase test conducted showed positive results for *Micrococcus* spp. and negative results for *Staphylococcus* spp. *Micrococcus* was oxidative while *Staphylococcus* was fermentative. We also used the colony pigmentation to differentiate between the two. *Staphylococcus* showed a typical white/light yellow color while *Micrococcus* showed an often bright yellow color.

These assays collectively served as confirmatory tests, facilitating the precise identification of the bacterial species under investigation. Germ tube test was also used to confirm the presence of *Candida* spp. from the yeast cells obtained after culturing.

### 2.4 Dipstick and Microscopy

Each urine specimen was mixed thoroughly; 5 mL was transferred into a 10 mL falcon tube. Subsequently, Medi‐Test Combi 10 SGL Urine Test Strips were used to conduct comprehensive biochemical assessments of each urine sample. Particularly, emphasis was placed on identifying leukocyte and nitrite. These parameters are indicative of potential bacterial infections. Further to this, the urine sample was centrifuged for 5 min at 1500 rpm. The supernatant was carefully decanted, and the residual material was used to prepare a wet mount for microscopic examination of the presence of pus cells by trained laboratory technicians (Rowe and Juthani‐Mehta 2014). All tests were read independently by at least two trained personnel, with a third reader involved to establish consensus in case of discrepancies.

### 2.5 Testing for Susceptibility to Antibiotics

According to the guidelines of the Clinical Laboratory Standards Institute, the Kirby Bauer disc diffusion method was used to screen each bacterial isolate for antibiotic susceptibility. This describes the standard zone of inhibition specific for each antibiotic. If the zone of inhibition observed was greater or similar to the standard inhibition zone, then the bacteria were considered sensitive to the antibiotic. On the other hand, if the detected zone of inhibition was smaller than the recommended size, the microorganism was considered resistant (Clinical Laboratory Standards Institute CLSI guidelines 2022). The selected antibiotics and their disc contents are as follows: levofloxacin (5 μg), ampicillin‐sulbactam (10 μg), cefotaxime (30 μg), ceftriaxone (30 μg), cefoperazone (30 μg), azithromycin (15 μg), cefuroxime (30 μg), chloramphenicol (30 μg), meropenem (10 μg), and tetracycline (30 µg). The antibiotics selected for susceptibility testing are the routine antibiotics used in clinical settings in Ghana for treating UTIs. The inclusion was also informed by known local resistance patterns from previous studies. (Donkor et al. 2019; Yevutsey et al. 2017).

### 2.6 Data Analysis

The data obtained were entered into Microsoft Excel 2021 and analyzed using the Statistical Package for Social Sciences (SPSS, software version 28). A descriptive analysis was used to summarize the study findings. Tabulated frequencies and corresponding percentages were employed for data presentation where appropriate. The association between socio‐demographic characteristics and UTI incidence was investigated using the chi‐square test. For all tests, *p* values less than 0.05 were considered statistically significant. Additionally, the Cohen's kappa coefficient (*κ*) was used to analyze the accuracy of the diagnostic tests. Briefly, *κ* < 0.20 indicates poor agreement, 0.21–0.40 fair, 0.41–0.60 moderate, 0.61–0.80 good, 0.81–0.99 very good and 1.00 indicates perfect agreement (McHugh 2012).

## 3 Results

### 3.1 Characteristics of Patients and UTI Status

Among the cohort of 208 patients included in this study, 48 (23.1%) were males, while 160 (76.9%) were females. Majority of the participants were aged between 50 and 59 years (36.5%) and married (61.5%). Most of the participants (54.8%) were traders, while only 3.4% were farmers. A total of 40.4% of participants had a secondary education, while 9.1% had no formal education. Overall, 48 out of 208 participants (23.1%; 95% CI: 17.6%–29.7%) were positive for UTIs using urine culture method as the standard tool (Table 1). Patients between the ages of 50 and 59 years (41.67%) recorded the highest prevalence of UTIs while those between the ages of 40 and 49 years recorded the least prevalence of 4.17%. A significant association was observed between age, gender, and the standard diagnostic method (*p* < 0.05), *p*‐value = 0.001 (Table 1).

**Table 1:** Socio‐demographic characteristics and UTI status of participants.

UTI status	Positive	Negative	Total	
Overall prevalence (culture)	48 (23.08%)	160 (76.92%)	208 (100%)	*p* value
**Age of participants (years)**				
18–29	15 (31.25%)	55 (34.38%)	70 (33.65%)	0.001
30–39	11 (22.92%)	26 (16.25%)	37 (17.79%)	
40–49	2 (4.17%)	23 (14.38%)	25 (12.02%)	
50–59	20 (41.67%)	56 (35.00%)	76 (36.54%)	
**Gender of participants**				
Male	2 (4.17%)	46 (28.75%)	48 (23.08%)	0.001
Female	46 (95.83%)	114 (71.25%)	160 (76.92%)	
**Marital status**				
Single	18 (37.50%)	62 (38.75%)	80 (38.46%)	0.876
Married	30 (62.50%)	98 (61.25%)	128 (61.54%)	
**Occupation**				
Farming	2 (4.17%)	5 (3.13%)	7 (3.37%)	0.253
Trading/business	25 (52.08%)	89 (55.63%)	114 (54.81%)	
Civil Servant	9 (18.75%)	37 (23.13%)	46 (22.12%)	
Student/Apprentice	5 (10.42%)	18 (11.25%)	23 (11.06%)	
Unemployed	7 (14.58%)	11 (6.88%)	18 (8.65%)	
**Highest level of education**				
Junior High School	12 (25.00%)	36 (22.50%)	48 (23.08%)	0.306
Senior High School	14 (29.17%)	70 (43.75%)	84 (40.38%)	
Tertiary Education	16 (33.33%)	41 (25.63%)	57 (27.40%)	
None	6 (12.50%)	13 (8.13%)	19 (9.13%)	

### 3.2 Prevalence of UTI According to the Three Diagnostic Methodologies

**Figure 1:**
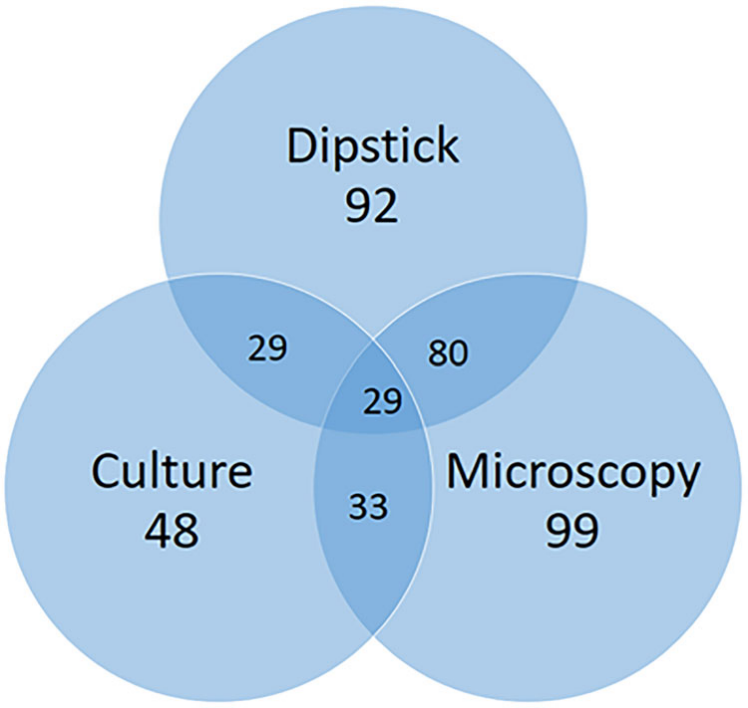
Prevalence of UTI according to the three diagnostic methods.

All 208 samples were tested for UTIs using the three diagnostic methods: dipstick, microscopy, and culture. A total of 92 (44.23%) samples tested positive for UTI using dipstick test while 99 (47.60%) samples tested positive using microscopy. For culture, a total of 48 (23.08%) samples tested positive for UTI (Figure 1). As shown in Figure 1, only 29 (13.9%) samples tested positive for UTI using all three diagnostic methods.

### 3.3 Comparison of Dipstick Test and Microscopic Examination Using Culture as Reference Standard

Using culture as the standard, dipstick test was more specific (60.62%) than microscopy (58.75%). Conversely, using culture as the standard, microscopy was more sensitive (68.75% vs. 60.42%), and reported higher positive (33.33% vs. 31.52%) and negative predictive values (86.24% vs. 83.62%) than the dipstick test as shown in Table 2. Furthermore, when assessing the concordance between microscopy and the dipstick test in relation to the standard diagnostic test, both techniques demonstrated a modest level of agreement with the standard. The Cohen's kappa coefficient for microscopy was determined to be *κ* = 0.200, while the dipstick test yielded a coefficient of *κ*= 0.159. Accordingly, microscopy recorded better diagnostic agreement with culture than the dipstick test for clinical investigation of UTIs.

**Table 2:** Diagnostic performance of microscopy and dipstick using culture as reference.

Performance metric	Test	
	Microscopy	Dipstick
TP (culture = 48)	33	29
FP (culture negative)	66	63
TN (culture = 160)	94	97
FN (culture positive)	15	19
Sensitivity % (95% CI)	68.75 (62.4–75.0)	60.42 (53.8–67.1)
Specificity % (95% CI)	58.75 (62.0–65.4)	60.62 (54.0–67.3)
PPV % (95% CI)	33.33 (26.9–39.7)	31.52 (25.2–37.8)
NPV % (95% CI)	86.24 (81.6–90.9)	83.62 (78.6–88.7)
Accuracy %	61.06	61.06
Kappa value (95% CI)	0.200 (0.084–0.317)	0.159 (0.037–0.282)

Abbreviations: FN, false negative; FP, false positive; NPV, negative predictive value; PPV, positive predictive value; TN, true negative; TP, true positive.

### 3.4 Microbial Isolates

**Figure 2:**
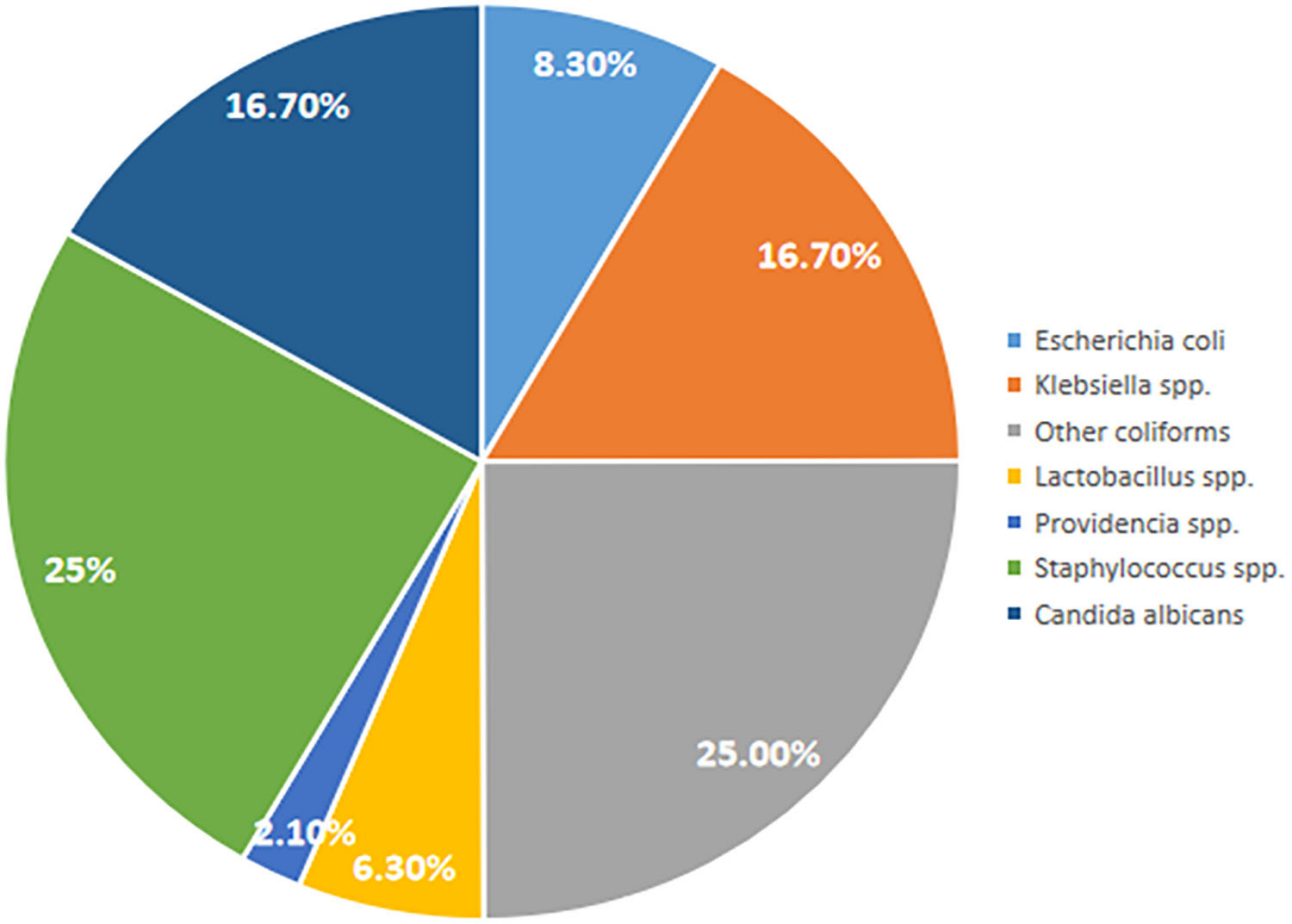
Distribution of microorganisms isolated from urine (*n* = 48).

Following microbiological analysis of urine samples from participants, the following bacteria were isolated: *E. coli*, *Klebsiella* spp., *Staphylococcus* spp., *Lactobacillus* spp., and *Providencia* spp. The only fungus species isolated was *Candida* spp. As shown in Figure 2, out of the 40 bacteria isolates, the most predominant Gram‐negative bacteria were *Klebsiella* spp. (16.7%). *Staphylococcus* spp. were the most predominant Gram‐positive bacteria (25%).

### 3.5 Antibiotics Resistance Pattern of Bacterial Isolates

**Figure 3:**
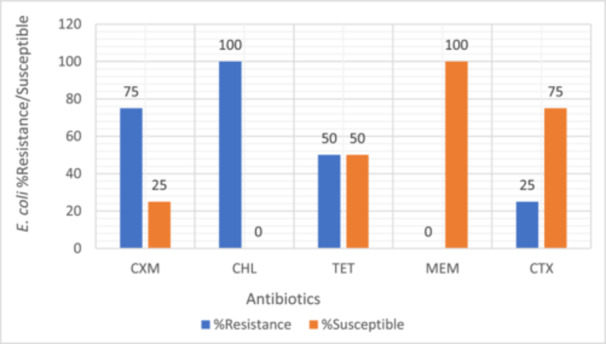
Antibiotic resistance and susceptibility pattern of *Escherichia coli* isolates (*n* = 4). CHL, chloramphenicol; CXM, cefuroxime; CTX, cefotaxime; MEM, meropenem; TET, tetracycline.

**Figure 4:**
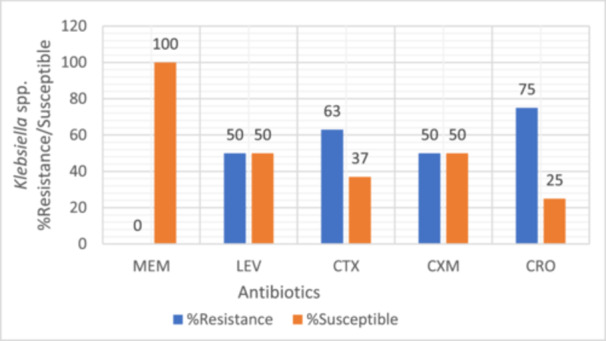
Antibiotic resistance and susceptibility pattern of *Klebsiella* spp. isolates (*n* = 8). CRO, ceftriaxone; CTX, cefotaxime; CXM, cefuroxime; LEV, levofloxacin; MEM, meropenem.

**Figure 5:**
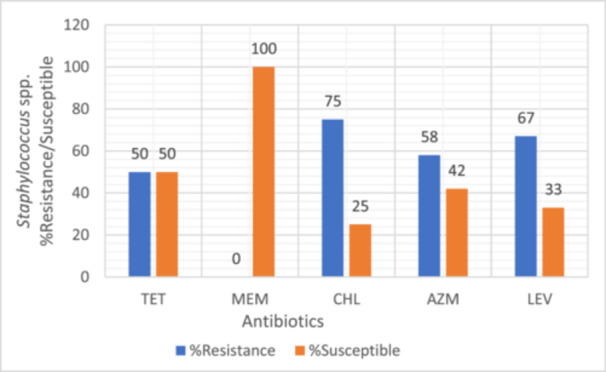
Antibiotics resistance susceptibility pattern of *Staphylococcus* spp. isolate (*n* = 12). AZM, azithromycin; CHL, chloramphenicol; LEV, levofloxacin; MEM, meropenem; TET, tetracycline.

**Figure 6:**
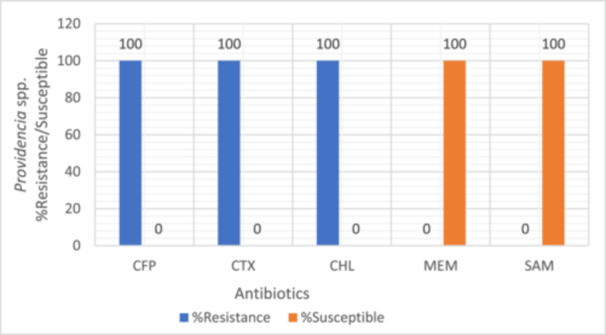
Antibiotic resistance and susceptibility pattern of *Providencia* spp. isolate (*n* = 1). CFP, cefoperazone; CHL, chloramphenicol; CTX, cefotaxime; MEM, meropenem; SAM, Ampicillin‐Sulbacam.

Bacterial isolates were evaluated for their susceptibility and resistance profiles against a range of antibiotics. Intermediate resistance results were treated as non‐susceptible and grouped together with resistant isolates for analysis as intermediate susceptibility often translates to reduced therapeutic efficacy in clinical settings, particularly in resource‐limited environments where optimized dosing or pharmacokinetic/pharmacodynamic monitoring may not be feasible. All tested bacterial strains were found to be susceptible to meropenem, as illustrated in Figures 3, 4, 5, 6. *E. coli, Klebsiella* spp., *Staphylococcus* spp., and *Providencia* spp. exhibited resistance to most of the antibiotics tested, with meropenem (MEM) remaining the exception.

Among the *E. coli* isolates, 75% exhibited resistance to cefuroxime (CXM), 100% to chloramphenicol (CHL), 50% to tetracycline (TET), and 25% to cefotaxime (CTX) (Figure 3). For *Klebsiella* spp., resistance was observed in 50% to levofloxacin (LEV), 63% to cefotaxime (CTX), 50% to cefuroxime (CXM), and 75% to ceftriaxone (CRO) (Figure 4).

In *Staphylococcus* spp., resistance rates were 50% to tetracycline (TET), 75% to chloramphenicol (CHL), 58% to azithromycin (AZM), and 67% to levofloxacin (LEV) (Figure 5). *Providencia* spp. showed complete resistance (100%) to cefepime (CFP), cefotaxime (CTX), and chloramphenicol (CHL) (Figure 6).

## 4 Discussion

The study sought to determine the prevalence and risk factors of UTIs among patients visiting a health facility. It also examined the performance of urinalysis as against culture in the diagnosis of UTI. In addition, the antibiotic susceptibility and resistance status of the isolated bacteria were determined.

The overall prevalence of UTI was 23.08% according to urine culture. This prevalence is similar to that in some sub‐Saharan Africa countries (Ayoyi et al. 2017) even though higher prevalences (Seifu and Gebissa 2018; Odoki et al. 2019) and lower prevalences have been recorded in some African countries (Dixon‐Umo 2020; Masika et al. 2017). However, in comparison to inpatients, higher prevalences have been observed (Lo et al. 2014; Akortha et al. 2012). Inpatients may have urethral catheters that predispose them to a greater risk of UTIs. Similarly, high prevalence (33.8%) has been observed among patients visiting hospitals in Northern Ghana (Karikari et al. 2022). In northern Ghana, water, sanitation and hygiene (WASH) conditions are challenging (World Health Organisation 2023). Conversely, other studies have observed lower UTI prevalence (10.1% and 15.9%) among some outpatients at Korle Bu and Mamprobi Polyclinics, and Ghana Police Hospital, respectively, both in Ghana's capital city, Accra (Donkor et al. 2019; Afriyie et al. 2015).

The observation that females exhibited a higher prevalence of UTIs in this study aligns with the findings of previous research efforts in Ghana and elsewhere (Iregbu and Nwajiobi‐Princewill 2013; Donkor et al. 2019; Karikari et al. 2022). This is mainly due to anatomical differences between the urethral of males and females. Other anatomical and physiological characteristics that make women more prone to acquiring UTIs are a drier urethral meatus, and a lack of prostatic fluid's antibacterial properties (Anuli et al. 2016). The age group of 50 to 59 years had the highest UTI prevalence in this study (41.67%); patients that fell within this age range were all females. These women are particularly vulnerable to developing both primary and recurrent UTIs due to biological changes associated with menopause. Again, the loss of estrogen weakens the walls of the urinary system, making them less resistant to bacterial colonization (Robinson et al. 2013).

The low concordance (13.9%) between dipstick/microscopy and culture results may be attributed to low bacterial loads in some urine samples that could result in false negatives on dipstick and microscopy, especially when bacterial concentrations fall below detection thresholds. Additionally, nonbacterial causes of pyuria or hematuria may lead to positive dipstick/microscopy results in the absence of culture‐confirmed infection. These findings underscore the limitations of relying solely on dipstick or microscopy for UTI diagnosis and highlight the need for culture confirmation, particularly in settings with high AMR. Compared to urine culture, dipstick method had sensitivity and specificity of 60.42% and 60.62%, respectively. Microscopy had a higher sensitivity value of 68.75% and a lower specificity of 58.75%. This suggests that while both dipstick and microscopy are good at correctly identifying individuals with UTIs (true positives), they can also misclassify healthy individuals as positive (false positives). This is critical in medical diagnostics since it could result in unnecessary treatments, cost, or anxiety, distress, and overall reduced trust among patients (Gupta et al. 2020). The result observed in this study is consistent with previous studies that showed similar sensitivities and specificities for microscopy and dipstick in diagnosing UTIs (Akin et al. 2011). Leukocytes may indicate an inflammatory reaction in the urinary system if they are seen in urine. In colonized as opposed to infected persons, the absence of an inflammatory response is associated with the presence of few or no leukocytes in urine. Additionally, patients with cell lysis may have false‐negative leukocyte counts (Bacârea et al. 2021; Akin et al. 2011).

While certain clinical symptoms can necessitate urinalysis, the symptoms should be used in addition to urine culture to diagnose UTI. The definitions of the nonspecific symptoms that many medical professionals have come to associate with UTI have lately evolved, even though many of the symptoms are evident (Brittany 2013). Although the dipstick method is quick, easy, and often preferred, its performance may be limited as it may produce false‐positive and false‐negative results. The test also needs the first urine sample produced in the morning since it takes bacteria 4 h to convert nitrate to nitrite at amounts that can be reliably detected (Kavuru et al. 2019).

In comparison to urine culture, wet preparation under the microscope has the advantage of quick turnaround time. However, microscopy requires extensive training, regular practice, and the appropriate tools (Kollerup et al. 2022). Leucocyturia alone may lead to false positives and some false negatives when used to detect culture positivity in patients without infection. False pyuria could be the result of contamination during sample collection, especially among female participants, noninfectious inflammatory conditions such as interstitial nephritis, certain sexually transmitted infections, particularly in women, delayed processing or poor preservation of urine samples which could lead to the degradation of bacteria cells while leukocytes remain detectable microscopically (Solh et al. 2017).

According to a report by Antwi et al. (2013), Gram‐negative bacteria accounted for more than 85% of all UTI infections and were the main etiologic agents of UTI among Ghanaian patients. In this study, 62.5% of identified bacteria were Gram‐negative. Previous studies have shown that the most predominant uropathogens included *E. coli* and *Klebsiella pneumoniae* (Iregbu and Nwajiobi‐Princewill 2013; Oladeinde et al. 2011; Battikhi and Battikhi 2015). In this study however, *Klebsiella* spp. and *Staphylococcus* spp. were the most predominant bacteria species identified. This could be the result of geographical and environmental variations including hygiene practices, water quality among others. Previous antibiotic exposure could also influence the etiology of UTI in our study (Asamoah et al. 2022; Afeke et al. 2025).

One of the most frequent organisms in nosocomial infections, *K. pneumoniae* has a propensity to develop multidrug resistance. This encapsulated Gram‐negative bacterium is a part of the typical oral, cutaneous, and intestinal flora (Li et al. 2014). *K. pneumoniae* UTI appears to be on the rise and has developed into a significant public health issue, particularly in hospital settings. In patients with chronic kidney disease, where urinary infections can accelerate the progression to end‐stage renal failure and are the second leading cause of death after cardiovascular issues, this trend must be addressed with even greater caution (Oana et al. 2017). *Klebsiella* spp. is frequently found in the human gut and other mucosal surfaces, and therefore, can easily colonize the urinary system, particularly in people who have certain risk factors like diabetes, a recurrent UTI, and those who have undergone recent antibiotic therapy (Lin et al. 2014). Recent studies however, point to an increase in the percentage of *Candida*‐caused UTI compared to UTI caused by bacteria particularly in hospitalized patients (Fisher et al. 2011; Bongomin et al. 2017; Gharanfoli et al. 2019; Gajdács et al. 2019). Urine containing *Candida* spp. may indicate contamination, colonization, UTI, or possibly candidemia (Pemán and Ruiz‐Gaitán 2018). *Staphylococcus* spp. was likewise shown to be the most common (5.8%) in related research by Amengialue et al. (2013). As a way to ascertain the frequency of *Staphylococcus* spp., a thorough community survey should be conducted as this may indicate that the organism was picked from the local area. *Staphylococcus* spp. is frequently linked to in‐patients who have invasive procedures like catheter implantation (Nor et al. 2013).

Most of the bacterial isolates were resistant to commonly used antibiotics such as levofloxacin, chloramphenicol, ceftriaxone, cefoperazone, cefuroxime, and cefotaxime. Meropenem was, however, highly effective in this study against the isolated bacteria. It could be considered for use in individuals with infections caused by problematic multi‐drug‐resistant bacteria, based on local susceptibility data. When used against both Gram‐negative and Gram‐positive bacteria, meropenem exhibited a wide range of activity. Meropenem was effective against the uropathogens, and should be used cautiously to preserve its efficacy. This result aligns with findings from other African countries, suggesting a regional drug‐resistance pattern (Maldonado‐Barragán et al. 2023; Merga Duffa et al. 2018; Koroma et al. 2019).

## 5 Conclusion

This study found a UTI prevalence of 23.1% among the 208 participants, with a significantly higher burden among females and individuals aged 50–59 years. Dipstick and microscopic urinalysis showed moderate sensitivity and specificity compared to culture, the gold standard, with microscopy demonstrating slightly better diagnostic concordance. Despite the established dominance of *E. coli* in UTI etiology, *Klebsiella* spp. and *Staphylococcus* spp. were more frequently isolated in this cohort. All bacterial isolates were susceptible to meropenem, while varying degrees of resistance were observed for commonly used antibiotics, highlighting the ongoing challenge of AMR. These findings underscore the importance of culture‐based diagnosis, continued surveillance of resistance patterns, and rational antibiotic use to inform effective UTI management strategies, particularly in resource‐limited settings. Limited funding constrained the study to a single site, limited some species‐specific biochemical tests, additional tests such as MALDI‐TOF/MS or molecular methods and prevented the molecular characterization of ESBL genes in the resistant isolates. The single‐center design employed in this study means that the data may not fully represent other regions with different epidemiological profiles or healthcare practices. Nonetheless, the results provide important insights into local trends and serve as a basis for future multi‐center studies.

## Author Contributions

**Michael Nana Antwi Boasiako:** conceptualization (lead), writing – original draft (lead), formal analysis (lead), writing – reviewing and editing (equal). **Kwadwo Boampong:** conceptualisation (supporting), writing – original draft (supporting), writing – reviewing and editing (equal). **Kingsley Badu:** conceptualisation (supporting), writing – original draft (supporting), writing – reviewing and editing (equal). **Frederick Ayensu:** conceptualisation (supporting), writing – reviewing and editing (equal). **Aaron Awuah:** writing – reviewing and editing (equal). **Abdul‐Hakim Mutala:** data analysis (supporting), writing – reviewing and editing (equal).

## Ethics Statement

Ethical approval was obtained from the Committee on Human Research Publications and Ethics (CHRPE) of the School of Medical Sciences, KNUST (CHRPE/AP/165/22). All the study participants signed informed consent forms after the study objectives were thoroughly explained to them in the local language where required.

## Conflicts of Interest

The authors declare no conflicts of interest.

## Data Availability

Data sharing is not applicable to this article as no data sets were generated or analyzed during the current study.
